# Physician Referral Patterns to Physical Therapists for Managing Knee Osteoarthritis: A Retrospective Analysis of Electronic Health Records From an Integrated Health System

**DOI:** 10.1002/acr.25630

**Published:** 2026-01-14

**Authors:** Samannaaz S. Khoja, Joel M. Stevans, Gustavo J. Almeida, Clair Smith, Janet K. Freburger

**Affiliations:** ^1^ University of Pittsburgh Pittsburgh Pennsylvania; ^2^ University of Texas Health Science Center at San Antonio

## Abstract

**Objective:**

This study aims to describe the frequency and timing of physician referrals to physical therapists (PTs) and other treatments prescribed over 12 months in patients with recent onset of knee osteoarthritis (KOA). The study also aims to identify determinants of early PT referrals.

**Methods:**

A retrospective study was conducted using electronic medical records from an integrated health care system in the United States. Incident KOA visits between October 2016 and September 2017 were identified using International Classification of Diseases, Tenth Revision codes. Early PT referral was defined as a referral within 15 days of the incident KOA visit by the attending physician. Data on PT referrals, other KOA treatments, patient characteristics, physician specialty, practice location, and volume of patients with KOA were extracted. The number of licensed PTs within the county of the included practices was also obtained. We used generalized linear mixed models to predict factors associated with early PT referrals.

**Results:**

Included in our study were 9,835 patients with incident KOA. Within the 12‐month period, 26% of patients had at least one PT referral (n = 2,550) and the median (interquartile range) days to the first referral was 0 (0–34) days. Early PT referral by the index physician occurred in 16.5% (n = 1,629) of patients, was less likely for patients with higher or missing knee pain scores and in rural practices, and more likely in women, patients with higher body mass index, and those seen in counties with a greater supply of licensed PTs.

**Conclusion:**

Physician referrals to PT for a new episode of KOA are infrequent and influenced by patient and practice factors.

## INTRODUCTION

Knee osteoarthritis (KOA) is a highly prevalent and costly chronic condition with no known cure. With increasing life expectancy and a growing aging population, the clinical and economic burdens associated with KOA‐related treatment and disability are expected to rise significantly in the coming decades.^1^ Although total knee arthroplasty (TKA) is the most effective treatment for end‐stage KOA, individuals may live several years with chronic knee pain and osteoarthritis‐related disability before undergoing TKA.

**SIGNIFICANCE & INNOVATIONS**

- Despite several clinical practice guidelines recommending nonpharmacologic treatments as frontline approaches for managing knee osteoarthritis (KOA), these interventions remain underused in clinical practice.
- This study provides valuable longitudinal data on the treatments recommended by physicians to patients with incident KOA over a 12‐month period, highlighting trends and gaps in care.
- Early physical therapist (PT) referrals were the most common nonpharmacologic approach that occurred in only 16.5% of the cohort identified with KOA. An additional 8% of the cohort with KOA received a PT referral after 15 days from the incident visit by their primary physician or another physician. Oral pain medications and therapeutic injections were more frequently prescribed compared with PT and lifestyle modifications.
- Early PT referrals were influenced by factors such as patient and practice characteristics, underscoring the need to continue addressing opportunities to improve the access and delivery of evidence‐based care for patients with KOA.

Since 1995, various clinical practice guidelines have recommended evidence‐based nonpharmacologic approaches such as exercise, weight management, and self‐management as frontline treatments, alongside nonsteroidal anti‐inflammatory drugs (NSAIDs) to manage and reduce pain and disability.^2^, ^3^, ^4^, ^5^, ^6^ These guidelines imply support for multidisciplinary teams to manage individuals with KOA, including physical therapists (PTs), who are uniquely qualified to provide exercise, self‐management counseling, and targeted treatments (eg, manual therapy, balance and functional exercises) to manage disability. However, the initial management of KOA in the United States is largely driven by physicians, with physician referrals being main route through which patients access physical therapy.

Despite updates in clinical practice guidelines, there remains a gap between current practice patterns and guideline‐based care. Specifically, physician referral to PTs tends to be suboptimal.^7^, ^8^, ^9^ Given that the guidelines recommend self‐management and exercise‐based interventions as frontline approaches for all individuals with KOA, we expect that referral to PT and other nonpharmacologic providers would be more common. Our previous work using the National Ambulatory Medical Care Survey showed a decline in PT referrals for KOA‐related orthopedic visits from 158 to 88 per 1,000 visits between 2007 to 2009 and 2013 to 2015, whereas referrals for KOA‐related primary care physician (PCP) visits remained low but steady, at 26 to 46 per 1,000 visits in the same period.^7^ In contrast, pain medication prescriptions for both NSAIDs (132–278 per 1,000 orthopedic visits; 221–498 per 1,000 PCP visits) and narcotics (77–236 per 1,000 orthopedic visits; 233–316 per 1,000 PCP visits) increased significantly within the same timeframe.^7^ Another study demonstrated similar findings with referral rates of 16% among all musculoskeletal disorders, including KOA,^10^ whereas one study from Australia indicated a lower PT referral rate of 5% for KOA.^9^ Additionally, the COVID‐19 pandemic has further contributed to a reduction in PT referrals for KOA.^11^

When referrals to PT remain low, the overall receipt of PT will remain low. Dhawan et al reported in a large cohort study using the 2004 to 2009 United Healthcare Database that only 25% of individuals with KOA received PT before TKA.^12^ Similarly, an analysis of the University of Utah health care medical records between from 2015 to 2018 showed that only 24% of patients referred to PT for knee pain actually used PT services.^10^ These low referral rates and low patient follow‐through to PT services coupled with increasing prescription rate for pain medications, including narcotics, suggest that those patients with KOA may not be receiving optimal, guideline‐based care.

Early treatment by PTs is associated with favorable patient outcomes and reductions in subsequent narcotics use, health care usage, and costs for knee pain.^13^, ^14^, ^15^, ^16^ However, current evidence suggests that most patients with KOA may not be adequately counseled about the benefits of PT, let alone receive a referral. Importantly, there is limited longitudinal evidence on the trajectory of care of individuals with incident KOA and factors that may influence the early referral of individuals with KOA to PT providers. It is likely that early referral to PT is not just driven by patient clinical characteristics but also by contextual factors surrounding their care. The Consolidated Framework for Implementation Research (CFIR) emphasizes the importance of examining contextual factors such as the inner settings (eg, clinic characteristics), outer setting (eg, supply of clinicians, insurance policies), and the individuals involved in health care delivery (eg, physicians) that may influence the uptake of evidence‐based approaches.^17^, ^18^ Hence, an understanding of contextual factors associated with PT referral for KOA at the patient, physician, and practice levels may help inform efforts to improve the use of PT for KOA. The aims of this study were to (1) describe the frequency and timing of physician referral to PT for patients with new‐onset KOA diagnosis; (2) describe the frequency of other KOA treatments or procedures prescribed within 12 months from the initial KOA‐related visit; and (3) identify patient, physician, and practice‐level determinants of early PT referral for KOA.

## PATIENTS AND METHODS

### Study design and data source

We conducted a retrospective cohort study using electronic medical records (EMRs) of patients seeking ambulatory care within a large, integrated health care system in western Pennsylvania. Limited EMR datasets were obtained from a research data warehouse^19^ via an honest broker following the establishment of a data use agreement with the health care system. The study was deemed exempt by the University of Pittsburgh Institutional Review Board.

We identified visits by patients who sought physician care for a new episode of KOA between October 1, 2016 and September 30, 2017 within one of the approximately 350 ambulatory care clinics within the system. A new episode was defined as an ambulatory care visit for KOA preceded by 12 months without any ambulatory care visits for KOA. We then gathered data on all subsequent KOA‐related procedures and treatments the patient received in the 12 months following the index physician visit (ie, between October 1, 2017 and September 30, 2018).

### Cohort identification and follow‐up

Patient records were included in the retrospective analysis if they (1) had an index visit for a new episode of KOA (defined as a visit between October 1, 2016 and September 30, 2017 related to KOA preceded by a clean period of 12 months with no KOA‐related visits), (2) were 45 years of age or older at the time of the index visit, and (3) KOA was recorded as the primary diagnosis, or as a secondary diagnosis with a related KOA procedure recorded at the visit (eg, knee x‐ray). The KOA diagnosis was identified using ICD‐10 (International Classification of Diseases, Tenth Revision) Clinical Modification codes (Supplementary Table 1). We used codes directly related to KOA (eg, M17X) as well as generic knee pain or swelling codes (eg, M25.56X) because KOA has shown to be underreported in the EMR and is usually coded as generic knee pain.^20^ Because we wanted to identify the earliest visit related to KOA, we included individuals with generic knee pain and swelling codes as their primary or secondary diagnosis, provided there were no other non‐KOA knee disorders, such as patellofemoral pain (ICD‐10 M22) or meniscal or ligament injuries (ICD‐10 M23), identified as a primary or secondary diagnosis at that visit. We did not exclude those with unilateral TKA if they were seeking care for their nonoperated knee but excluded those with evidence of bilateral TKA (ICD‐10 Z96.653, V43.65) before their initial KOA visit.

### Dependent variable

Our primary dependent variable “early referral to PT” was defined as a PT referral provided by the index physician at the index KOA visit or within 15 days of that visit.^13^ We also collected data on PT referrals between 16 and 365 days from the index visit. PT referrals were identified via internal custom codes from the orders field in the EMR (Supplementary Table 2).

To describe downstream KOA care over a 12‐month period in our cohort, we collected data on referrals to lifestyle or exercise counseling consults, referrals to specialty physicians (ie, orthopedists, physical medicine and rehabilitation, sports medicine), knee‐related tests or procedures obtained (ie, therapeutic injections, imaging, surgery), and pain medications prescribed (topical and oral NSAIDs, tramadol, and nontramadol narcotics). Supplementary Tables 2 to 4 lists the downstream treatments and procedures that were collected. These data were collected from the index visit through the 12‐month follow‐up period for each patient. All downstream health care services associated with a KOA diagnosis for each patient were extracted regardless of the clinician providing the services.

### Explanatory variables

Selection of our independent variables of interest was guided by our previous work and reported literature regarding the barriers and facilitators related to health care processes and decisions.^7^, ^21^ We hypothesized that factors related to the health care environment such as practice characteristics related to the volume of patients with KOA (inner setting), practice location and supply of licensed PTs (outer settings), individuals involved in providing health care (ie, physician), and recipients of care (ie, patients with KOA) would be associated with the likelihood of receiving a PT referral from a physician. We used the CFIR to inform the selection of our explanatory variables from the EMR (Supplementary Table 5).^17^

Physician characteristics available in the EMR included physician specialty and their volume of patients with KOA seen. The volume of patients with KOA was defined as the total number of identified patients with KOA (within the study period, ie, between October 1, 2016 to September 30, 2018) managed by each physician within our dataset. We hypothesized that physicians with a higher volume of patients with KOA would have different referral patterns or KOA management practices compared with those with a lower volume of patients with KOA. Practice characteristics included urban or rural location based on the designation of the county where the practices were located;^22^ availability of PTs within the county where the practice was located, calculated as the number of registered licensed PTs per 10,000 county residents,^23^ and volume of patients with KOA at the practice level, calculated as the percentage of patients with KOA relative to the total number of patients seen in the practices during the study data collection period.

Patient characteristics extracted from the EMR included demographic information (ie, age, sex, self‐reported race and ethnicity, and insurance) and clinical information (ie, body mass index, smoking status, presence of comorbid conditions, and pain levels). To capture comorbid conditions, we calculated an Elixhauser summary score for each patient based on the associated ICD‐10 diagnoses present in their EMR data.^24^, ^25^

### Data extraction and statistical analysis

Raw data files with the limited dataset were obtained by the honest broker and then processed to remove duplicates and noneligible encounters (nonphysician visits, no KOA‐related diagnoses, encounter outside of the study period, missing information on index physician specialty). Variables of interest were identified via structured data fields such as Current Procedural Terminology codes and internal custom codes (Supplementary Tables 2–4). We reformatted or collapsed categories for variables (eg, insurance type, provider specialty, smoking status) as appropriate. A clean analytic file was then exported to the SAS statistical package for statistical analysis.

We used descriptive statistics to characterize patient, physician, and practice characteristics, to calculate the proportion (%) of patients who received a PT referral (within 15 days of index visit or after) and over 12 months, and to summarize the distribution of time to receipt of first PT referral. Similarly, we used descriptive statistics (frequency and proportions) to describe other KOA referrals, treatments, and procedures at the index visit and over the 12‐month period.

We used generalized linear mixed models (GLMMs) to examine predictors of early referral to PT. The models had a logit link and practice as the random intercept to control for clustered design of the study (patients are clustered by practice). We assessed for multicollinearity among our predictor variables with the phi correlation coefficient. If two variables were highly correlated (ie, coefficient > 0.8) we made decisions to drop one or the other for methodologic (eg, missingness) or conceptual reasons. We then used backward elimination to retain variables that were significant at *P* < 0.05). For observations with missing data among the predictor variables, we created an additional “missing” category for those predictors and included them in the model. We conducted one sensitivity analysis to examine whether determinants of early PT referral differed among those with ICD‐10 codes specific to KOA compared with those with only general knee symptoms codes. Analyses were done using SAS (version 9.4) software.

## RESULTS

We identified 10,101 eligible patients with a primary or secondary diagnosis related to KOA from a total of 27,160 patient records with an ambulatory care visit performed by a physician during the study period. Patient records were excluded if they were duplicative (ie, for a patient with more than one eligible visit only the earliest visit was retained), missing provider specialty information, age less than 45 years at index visit, visit date beyond data collection period, or had a noneligible knee diagnosis. Additionally, patients were excluded from the final analytic dataset if the practice location was unknown or missing, or if they were seen in practices with five or fewer total patients with KOA. Because practice was determined to be a random effect, we excluded practices with five or fewer observations due to model convergence issues. See Supplementary Figure 1 for list of cohort exclusions. The final analytic dataset included 9,835 patients with KOA.

Overall, the sample was older than 60 years of age, mostly women (62%), non‐Hispanic White (87%), and had an average body mass index (BMI) in the obese category (>30). Most patients were seen by a PCP or orthopedist at the index visit. Table 1 describes sample characteristics based on whether early PT referral was received or not. Missing data were observed for pain, BMI, and volume of patients with KOA per physician and per practice. For the practice‐level volume of patients with KOA, the missingness was less than 1%; thus, we did not create a separate category. For the other three variables (ie, pain, BMI, and volume of patients with KOA per physician), we created a “missing” category and included it in the model to account for the incomplete data.

**Table 1:** Characteristics of the cohort with KOA*

Characteristics	Early PT referral (n = 1,629)	PT referral beyond 15 days or no referral (n = 8,206)
Patient characteristics		
Age, mean ± SD, y	63.7 ± 10.5	65.1 ± 11.1
Age category, n (%), y		
45–59	590 (36)	2,670 (33)
59–69	562 (34)	2,779 (34)
>69	477 (29)	2,757 (34)
BMI, mean ± SD	31.9 ± 7.4	31.9 ± 7.3
BMI category, n (%)		
<25	243 (15)	1,119 (14)
25–30	435 (27)	2,199 (27)
>30	805 (49)	4,005 (49)
Missing	146 (9)	883 (11)
Female sex, n (%)	1,096 (67)	4,960 (60)
Race, n (%)		
White	1,392 (85)	7,412 (90)
Black or African American	186 (11)	637 (8)
Other, declined, or unknown^a^	51 (3)	157 (2)
Ethnicity, n (%)		
Hispanic or Latino	9 (1)	55 (1)
Not Hispanic or Latino	1,558 (96)	7,933 (97)
Declined or unknown	62 (4)	218 (3)
Insurance type, n (%)		
Commercial or private	960 (59)	4,680 (57)
Medicaid	140 (9)	635 (8)
Medicare	503 (31)	2,783 (34)
Other	26 (2)	108 (1)
Smoking status, n (%)		
Never smoker	893 (55)	4,262 (52)
Current smoker	172 (11)	944 (12)
Former smoker	561 (3)	2,987 (36)
Unknown	3 (0)	13 (0)
Pain, mean ± SD, numeric pain scale (0–10)	5.5 ± 2.6	6 ± 2.5
Pain category, n (%)		
0–3	165 (10)	439 (5)
4–6	255 (16)	942 (11)
7–10	275 (17)	1,102 (13)
Missing pain scores	934 (57)	5,723 (70)
Practice characteristics		
Rural practice location, n (%)	312 (19)	2,671 (33)
County‐level number of PTs per 10,000 residents, mean ± SD	11.4 ± 1.8	10.8 ± 2.2
Number of PTs per 10,000 residents, n (%)		
<10 PTs per 10,000 residents	215 (13)	2,031 (25)
>10 PTs per 10,000 residents	1,414 (87)	6,175 (75)
Physician characteristics		
Physician specialty, n (%)		
Orthopedic surgeon	892 (55)	3,168 (39)
Primary care physician	610 (37)	4,340 (53)
Other	127 (8)	698 (9)
Volume of patients with KOA, n (%)		
0–14	505 (31)	2,986 (36)
15–79	395 (24)	2,693 (33)
>80	725 (45)	2,495 (30)
Missing	4 (0)	32 (0)
Patients with KOA as a proportion to total practice volume, n (%)		
≤2.3	190 (12)	1,455 (18)
2.4–3.7	236 (14)	1,586 (19)
≥3.8	230 (14)	1,362 (17)
Missing	973 (60)	3,803 (46)
Elixhauser Comorbidity index, n (%)		
0 or 1 comorbidity	494 (30)	2,341 (29)
2 or 3 comorbidities	529 (32)	2,669 (33)
4 or 5 comorbidities	338 (21)	1,675 (20)
≥6 comorbidities	268 (16)	1,521 (19)

* BMI, body mass index; EMR, electronic medical record; KOA, knee osteoarthritis; PT, physical therapist.

^a^ Includes race categories of American Indian or Alaskan Native, Native Hawaiian or other pacific islander, Asian, multiracial origin, and if declined or not reported in the EMR.

**Figure 1:**
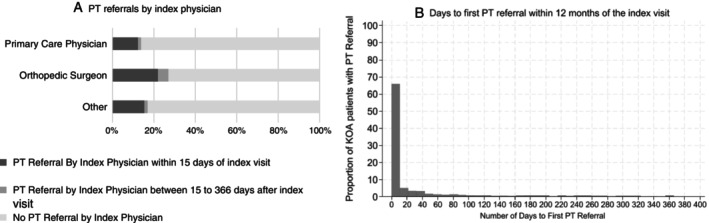
Rate and timing of the first PT referral received in the cohort with KOA over the 12‐month follow‐up period. KOA, knee osteoarthritis; PT, physical therapist.

Of the 9,835 patients included in this analysis, 16.5% (n = 1,629) received an early PT referral from their index physician. Orthopedists had the largest proportion of patients receiving early PT referral (Table 1 and Figure 1A), and most PT referrals (n = 1,513) were provided at the index visit (Figure 1B). A small proportion of the cohort received PT referrals within 15 days (1.3%, n = 127) or beyond 15 days (5.1%, n = 502) from that initial visit from providers other than their index physician. Additionally, a small proportion of the cohort received a PT referral from their index physician between 16 and 365 days from their initial visit (~3%, n = 293). The total number of PT referrals per patient within the 12‐month period in the cohort with KOA ranged from zero to six referrals; around 73% of patients had zero PT referrals, 21% had at least one PT referral, and 5% had two or more PT referrals. The median (interquartile range) number of days to the first PT referral by any physician after the index visit was 0 (0–34) days. The rate of early PT referral was also similar when looking at patients based on diagnostic category. About 38% of patients identified had a KOA‐specific diagnosis, whereas 62% had only general knee symptoms. Early PT referral was observed in 16.7% of patients with a KOA‐specific diagnosis and in 16.3% of patients with only general knee symptoms (Supplementary Table 6).

**Figure 2:**
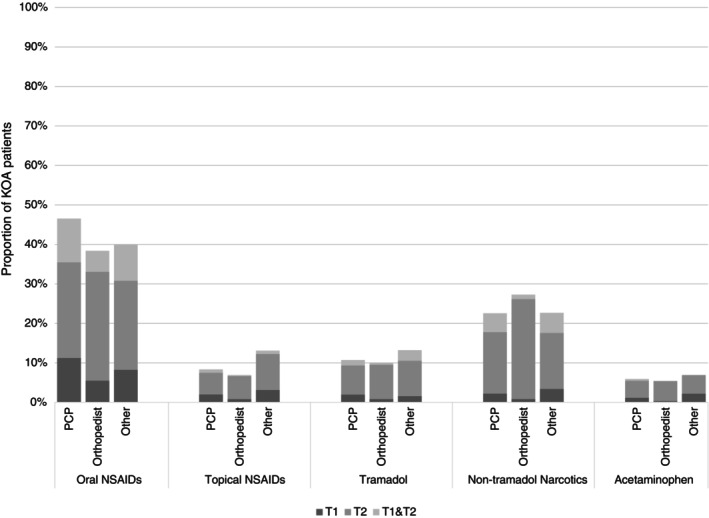
Proportion of patients who were prescribed medications for KOA at index visit (T1), or at a follow‐up visit within 12 months of the index visit (T2), or at both index and follow‐up visits (T1 and T2). KOA, knee osteoarthritis; NSAIDs, nonsteroidal anti‐inflammatory drugs; PCP, primary care physician.

**Figure 3:**
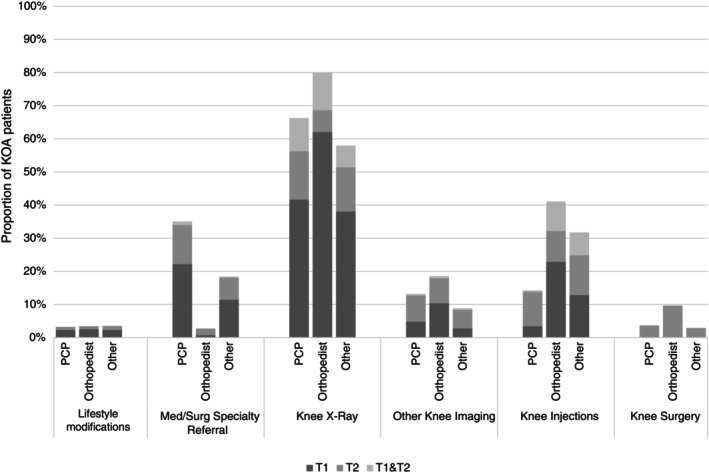
Proportion of patients who received treatments or procedures for KOA at index visit (T1), or at a follow‐up visit within 12 months of the index visit (T2), or at both index and follow‐up visits (T1 and T2). KOA, knee osteoarthritis; Med/Surg, medical or surgical; PCP, primary care physician.

Other referrals, procedures and medications prescribed during the 12‐month follow‐up are reported in Figures 2 and 3. Around 20% of patients with KOA received referrals to other medical or surgical physician specialties. The most common procedure was knee x‐ray (~70%), followed by therapeutic injections (~26%). The most common type of medications prescribed were NSAIDs (~41%) followed by nontramadol narcotics (~25%).

The multicollinearity analysis revealed only one significant relationship among the predictor variables: physician specialty and physician volume of patients with KOA. We retained physician specialty in the model because it had no missing values, whereas physician volume of patients with KOA had a small percentage (<1%) of missing values. The results of the GLMM analysis are presented in Tables 2 and 3. Among patient characteristics, women were more likely to receive an early PT referral, whereas patients with missing or higher pain scores were less likely to receive an early PT referral. BMI as a category was significantly associated with early PT referral; however, the odds ratios of individual categories (overweight, obese, or missing) were not significantly different from the reference group (BMI < 25). Physician specialty was not significantly associated with the likelihood of receiving an early PT referral. Among practice characteristics, location and supply of PTs were significantly associated with the receipt of PT referral. Patients seen in rural practices were less likely to receive an early PT referral, and those seen in counties with greater PT availability were more likely to receive an early PT referral. Sensitivity analysis based on KOA diagnostic category did not reveal any major differences in the magnitude or direction of associations between our dependent variable and the predictor variables when compared to the full model (Supplementary Tables 7 and 8). Briefly, sensitivity analysis did reveal a stronger magnitude of association between PT availability and location with early PT referral in the KOA‐specific diagnosis subcohort, whereas association between BMI and early PT referral was stronger in the general knee symptom subcohort.

**Table 2:** Full model showing univariate associations between patient, physician, and practice factors and early PT referral*

Independent variables	OR (95% CI)	*P* value
Patient characteristics		
Age, y		
45–59 (ref)	1.00	0.136
59–69	0.93 (0.80–1.07)	
>69	0.84 (0.71–1.00)	
Sex		
Male (ref)	1.00	**<0.0001**
Female	1.43 (1.27–1.62)	
Race		
White (ref)	1.00	0.221
Black	1.11 (0.88–1.38)	
Other	1.32 (0.92–1.89)	
Ethnicity		
Not Hispanic or Latino (ref)	1.00	0.349
Hispanic or Latino	0.66 (0.31–1.41)	
Declined or missing	1.17 (0.85–1.60)	
Health insurance coverage		
Commercial (ref)	1.00	0.256
Medicaid	1.07 (0.85–1.35)	
Medicare	1.13 (0.98–1.30)	
Other	1.32 (0.82–2.12)	
BMI		
<25 (ref)	1.00	**0.024**
25–30	1.06 (0.88–1.28)	
>30	1.13 (0.95–1.35)	
Missing	0.78 (0.60–1.03)	
Smoking status		
Current smoker (ref)	1.00	0.820
Former smoker	1.05 (0.85–1.29)	
Never smoker	1.09 (0.89–1.33)	
Unknown	1.42 (0.35–5.78)	
Pain category, numeric pain scale (0–10)		
0–3 (ref)	1.00	**0.0004**
4–6	0.76 (0.59–0.97)	
7–10	0.71 (0.55–0.91)	
Missing	0.60 (0.47–0.76)	
Elixhauser Comorbidity index		
0 or 1 comorbidity (ref)	1.00	0.306
2 or 3 comorbidities	0.95 (0.82–1.11)	
4 or 5 comorbidities	0.96 (0.81–1.15)	
≥6 comorbidities	0.84 (0.69–1.01)	
Physician characteristics		
Specialty		
PCP (ref)	1.00	0.683
Orthopedics	1.09 (0.69–1.72)	
Other	1.17 (0.82–1.67)	
Practice characteristics		
PT availability		
<10 PTs per 10,000 residents (ref)	1.00	0.081
>10 PTs per 10,000 residents	1.39 (0.96–2.02)	
Location		
Urban (ref)	1.00	**0.013**
Rural	0.64 (0.46–0.91)	

* Bold values denote significance at *P* < 0.05. BMI, body mass index; CI, confidence interval; OR, odds ratio; PCP, primary care physician; PT, physical therapist; ref, reference group.

**Table 3:** Final model with multivariable associations between patient, physician, and practice factors and early PT referral*

Independent variables	OR (95% CI)	*P* value
Patient characteristics		
Sex		
Male	1.00	<0.0001
Female	1.43 (1.26–1.61)	
BMI		
<25	1.00	
25–30	1.06 (0.88–1.27)	0.019
>30	1.13 (0.95–1.34)	
Missing	0.78 (0.59–1.02)	
Pain category, numeric pain scale (0–10)		
0–3	1.00	0.0002
4–6	0.76 (0.59–0.97)	
7–10	0.70 (0.54–0.89)	
Missing	0.59 (0.46–0.75)	
Practice characteristics		
PT availability		
<10 PTs per 10,000 residents	1.00	0.048
>10 PTs per 10,000 residents	1.43 (1.00–2.04)	
Location		
Urban	1.00	
Rural	0.65 (0.46–0.91)	0.013

* BMI, body mass index; CI, confidence interval; OR, odds ratio; PT, physical therapist.

## DISCUSSION

Our study describes the care that patients with incident KOA received over a 12‐month period in a large integrated health system. It also examines factors at the patient, practice, and physician levels that are associated with receipt of early PT referrals. We found a low rate of PT referrals for KOA (16.5%), consistent with previous reports that have reported PT referral rates for KOA between 5 and 16%.^9^, ^10^, ^12^ Early PT referrals were most common when the index physicians were orthopedic surgeons compared with PCPs or other specialists. Some possible explanations include orthopedic surgeons having greater experience with rehabilitation approaches and guideline‐based care for KOA, the patient not being a candidate for surgery, or the patient needing conservative management before considering surgery.

A unique finding from our study pertains to the timing of PT referrals. Our results indicate that over 50% of PT referrals occurred at the index visit and up to 75% of them occurred within 34 days of that index visit. Further, Figure 1B shows a sharp decline in PT referrals around two weeks from the index visit. Among patients who received a PT referral, most were referred only once, either by the index physician or by another physician within the 12‐month follow‐up period. These observations indicate that PT referrals tend to occur early in the management of KOA and are rarely reconsidered later in the 12‐month care trajectory. This pattern highlights a potential missed opportunity to incorporate PT as a core component of ongoing conservative care for KOA. Considering PT early on during the management of KOA is critical due to its impact on patient outcomes and downstream health care use. Previous studies have indicated that early PT initiation has implications for lowering risk related to the use of opioids,^13^, ^15^ intra‐articular injections,^13^, ^26^ and knee surgery.^13^, ^27^

Our findings align with previous studies that have shown that nonpharmacologic approaches are underused relative to pharmacological approaches for KOA.^9^, ^28^ PT referral was the most frequent nonpharmacologic approach (16.5% of patients), whereas lifestyle modifications were documented in only 0.3% of patients and referrals for complementary and alternative medicine were nearly nonexistent in our dataset. Although not all patients with KOA require a PT referral, we would expect that most would be educated about exercise, lifestyle, and self‐management interventions because they are strongly recommended in recent clinical practice guidelines.^2^, ^6^ The infrequent use of lifestyle modifications and complementary and alternative medicine in our data could be multifactorial but not limited to physician knowledge, lack of defined referral resources, and inadequate EMR documentation. Relative to nonpharmacologic approaches, use of pain medications (NSAIDs, 41% of patients; nontramadol narcotics, 25% of patients) including therapeutic injections (26% of patients) was higher. Although use of NSAIDs is guideline‐concordant, the use of nontramadol narcotics during early KOA management is concerning because the most recent American College of Rheumatology and Arthritis Foundation guidelines conditionally recommend against their use.^2^ Therapeutic injections may be appropriate; however, past evidence suggests that therapeutic injections do not have superior benefits over exercise or PT interventions.^14^, ^29^ A recent meta‐analysis also indicated a potential superior benefit of exercise over NSAIDs, although there is a paucity of trials directly comparing efficacy of pharmacologic versus nonpharmacologic approaches for patients with KOA.^30^ Despite their comparable or superior benefits relative to pharmacological approaches, PT or exercise is underprescribed as a frontline approach for patients with KOA.

The final multivariable model identified three patient characteristics and two practice characteristics that were significantly associated with early PT referrals. Among patient characteristics, the reduced likelihood of PT referrals in patients with higher knee pain scores may reflect the preference of physicians and patients for interventions that can be provided immediately at that same visit such as medications or injections to address symptoms before considering PT, or could be driven by missing data and bias in data collection of pain scores. Women were more likely to receive an early PT referral, and this finding aligns with prior research that suggests that women are more likely to use physical therapy.^31^, ^32^ With respect to BMI, although no individual categories were significantly different from the referent category, the odds ratios suggest a general trend for individuals with greater BMI to have a higher likelihood of receiving an early PT referral. This may reflect the physician's perception of PT being a higher priority for individuals with higher BMI due to the known association of obesity with KOA symptoms, the overall benefits of exercise for obesity, and potential need to reduce weight before TKA.

In terms of practice characteristics, patients seen in rural practices were less likely to receive early PT referrals compared with those seen in urban practices, and those seen in counties with more than 10 licensed PTs per 10,000 residents were more likely to receive an early referral. These findings reflect geographic disparities in care due to decreased accessibility of resources including supply of PTs. Reduced accessibility to PT services in rural areas has been reported in the literature^7^ and is acknowledged as an important factor contributing to health care disparities. Geographic neighborhood‐level deprivation has been associated with worse KOA symptoms and outcomes, especially after TKA.^33^, ^34^ One recent publication in the French health care system reported lower likelihood PT referral by physicians for musculoskeletal disorders in areas with higher deprivation index.^35^ To date, the relationship between early PT referral for patients with KOA and geographic or social deprivation has not been extensively studied and future studies on this topic are warranted to minimize disparities in care and improve accessibility for those with KOA.

The findings of this study suggest that the referral to PT for conservative care of patients with KOA is relatively low and is influenced by factors at the patient, physician, and practice levels. Although we did not assess whether patients who received a PT referral attended PT; we anticipate that this proportion would be even lower, indicating that patients who may benefit from PT are not receiving PT. The study also illustrates the importance of continued investigation and investment in implementing alternative models of care to improve access and use of PT, increase patients’ engagement in nonpharmacologic interventions (eg, exercise and lifestyle changes), and improve physician adherence to KOA guideline recommendations. Some examples of KOA models of care being examined to improve access and use of KOA guideline‐recommended treatments include telerehabilitation^36^, ^37^, ^38^, ^39^, ^40^ and interdisciplinary osteoarthritis programs that include a PT referral or visit defined within the care pathway.^41^

Our study is not without limitations. Because we had an observational study using EMR data, it is subject to inherent errors and missing data commonly associated with EMR documentation. There is a possibility that we may have misclassified patients with KOA because we included ICD‐10 codes for patients with generic knee pain or symptoms without any other ICD‐10 codes indicating alternative pathologies and those with evidence of unilateral TKA seeking care for their nonoperated knee. Although individuals with unilateral TKA are likely to have a different trajectory of care due to their experience with KOA compared with those without any knee replacement surgery, the number of such patients in our sample were quite small (~2%) and likely not to impact the overall findings. We acknowledge that some of our findings may be due to sampling bias, missing data, or unique characteristics of patients within our health system compared with the general US population with KOA. The sampled cohort with KOA, although representative of patients in our health care system, was not very diverse (non‐White non‐Black racial and ethnic groups were < 2%) and does not reflect the population with KOA in the United States. Additionally, only EMR data were available for this study, which limited our ability to examine other recommended CFIR contextual factors such as the influence of patient resources (eg, copay burden, time constraints) and preferences or perceptions about PT (previous PT experience, misconceptions about exercise and osteoarthritis), and physician perceptions about benefits of PT for patients with KOA. This study is also focused on physician behavior (ie, providing a PT referral) and does not account for whether the patient attended a PT session.

Despite these limitations, our study has some notable strengths. It includes a large, representative sample of patients from a major health care system in western Pennsylvania. Also, we captured longitudinal data to assess how patients were managed for one year from their first identifiable visit related to a new episode of care for KOA symptoms.

This study demonstrated that the rate of physician referrals to PT for a new episode of KOA is low, and when present, typically occurs at the incident visit. Early physician referral to PT was influenced by few patient factors (female sex, level of pain, BMI) and by practice factors (urban or rural location). More commonly used KOA treatments over 12‐month period from the index visit included therapeutic injections or pharmacological agents.

## AUTHOR CONTRIBUTIONS

All authors contributed to at least one of the following manuscript preparation roles: conceptualization AND/OR methodology, software, investigation, formal analysis, data curation, visualization, and validation AND drafting or reviewing/editing the final draft. As corresponding author, Dr Khoja confirms that all authors have provided the final approval of the version to be published and takes responsibility for the affirmations regarding article submission (eg, not under consideration by another journal), the integrity of the data presented, and the statements regarding compliance with institutional review board/Declaration of Helsinki requirements.

## Supporting information


**Disclosure form**.

**Appendix S1:** Supplementary Information.
