# Supplementary material for: Physician Referral Patterns to Physical Therapists for Managing Knee Osteoarthritis: A Retrospective Analysis of Electronic Health Records From an Integrated Health System
Source: Arthritis Care Res (Hoboken). 2026 Jan 14;78(4):478–88. doi: 10.1002/acr.25630 (PMC13034102; doi:10.1002/acr.25630)
Supplement: Supplementary file 2 — Appendix S1: Supplementary Information. [file ACR-78-478-s002.docx]

**Supplementary Documents, Tables and Figures**

Table of Contents:

1. Summary of data extraction procedures
2. Supplementary Table S1 – ICD10-CM diagnostic codes to identify KOA related ambulatory visits
3. Supplementary Figure S1 – Cohort identification flow chart
4. Supplementary Table S2 – Referral or consult codes from the EMR
5. Supplementary Table S3 – Medication descriptions from the EMR
6. Supplementary Table S4 – Procedure and treatment codes from the EMR
7. Supplementary Table S5 – Study Variables mapped to the Consolidated Framework for Implementation Research (CFIR)
8. Supplementary Table S6 – Number and percentage of patients that received an early PT referral by diagnosis category
9. Supplementary Table S7– Sensitivity Analysis for Early referral to PT: Subset of cohort with specific KOA diagnostic code at the index visit
10. Supplementary Table S8 – Sensitivity Analysis for Early referral to PT: Subset of cohort with only general knee symptoms codes at the index visit

**Data extraction summary of procedures:**

A limited dataset with all variables of interest was obtained by the honest broker in several flat comma separated values (CSV) files. The honest broker stripped the data of protected health information and provided a study identifier (ID) for each unique patient in the cohort prior to sharing the data with the study team. A data analyst with programming experience pre-processed these raw files using Microsoft SQL to remove duplicates and non-eligible encounters (non-physician providers, no KOA related diagnoses, encounter happened outside of the study period, data on index physician specialty was missing). Primary and secondary variables of interest were identified via structured data fields such as CPT codes and internal custom codes (Tables 2, 3, 4). Once eligible encounters were identified, an analytic dataset with the final cohort and variables of interest was created using MS SQL. Additional cleanup of the analytic dataset including reformatting or collapsing categories for some variables of interest (e.g., insurance type, provider specialty, smoking status) was done as needed to create the final analytic dataset.

**Supplementary Table S1. ICD10-CM diagnostic codes used to identify knee-osteoarthritis related ambulatory visits (office encounters):**

| M17 Osteoarthritis of knee |
| --- |
| M17.0 Bilateral primary osteoarthritis of knee |
| M17.1 Unilateral primary osteoarthritis of knee |
| M17.10 Unilateral primary osteoarthritis, unspecified knee |
| M17.11 Unilateral primary osteoarthritis, right knee |
| M17.12 Unilateral primary osteoarthritis, left knee |
| M17.2 Bilateral post-traumatic osteoarthritis of knee |
| M17.3 Unilateral post-traumatic osteoarthritis of knee |
| M17.30 Unilateral post-traumatic osteoarthritis, unspecified knee |
| M17.31 Unilateral post-traumatic osteoarthritis, right knee |
| M17.32 Unilateral post-traumatic osteoarthritis, left knee |
| M17.4 Other bilateral secondary osteoarthritis of knee |
| M17.5 Other unilateral secondary osteoarthritis of knee |
| M17.9 Osteoarthritis of knee, unspecified |
| M24.56 Contracture, knee |
| M24.561 Contracture, right knee |
| M24.562 Contracture, left knee |
| M24.569 Contracture, unspecified knee |
| M25.46 Effusion, knee |
| M25.461 Effusion, right knee |
| M25.462 Effusion, left knee |
| M25.469 Effusion, unspecified knee |
| M25.56 Pain in knee |
| M25.561 Pain in right knee |
| M25.562 Pain in left knee |
| M25.569 Pain in unspecified knee |
| M25.66 Stiffness of knee, not elsewhere classified |
| M25.661 Stiffness of right knee, not elsewhere classified |
| M25.662 Stiffness of left knee, not elsewhere classified |
| M25.669 Stiffness of unspecified knee, not elsewhere classified |
| M25.76 Osteophyte, knee |
| M25.761 Osteophyte, right knee |
| M25.762 Osteophyte, left knee |
| M25.769 Osteophyte, unspecified knee |

**Supplementary Figure S1. Flow Diagram on Cohort Identification**

Exclusions:

6 – age <45, missed earlier due to rounding error calculating age

15 – unknown/missing practice location

245 – seen in practices with <5 KOA patients

Exclusions:

682 – missing provider specialty

309 – age <45 years

115 – duplicative patient IDs with more than one eligible KOA visit

37 – more than one of the above exclusions.

643 – index visit date beyond 9/30/2017

291 – other KOA diagnosis - M22, M23

Exclusions:

14,982 - no associated primary or secondary KOA diagnosis at the visit

**Supplementary Table S2. KOA related referral or consults identified in the Electronic Medical Records with proprietary codes that are not generalizable.**

| **Description of the code** | **Final category reported in manuscript** |
| --- | --- |
| CONSULT / REFERRAL TO PHYSICAL THERAPY | PT Referral |
| CONSULT / REFERRAL TO AQUATIC PROGRAM | PT Referral |
| CONSULT / REFERRAL TO PHYSICAL THERAPY FOR CONTINUING CARE | PT Referral |
| CONSULT / REFERRAL TO AQUA THERAPY | PT Referral |
| CONSULT / REFERRAL TO BALANCE TRAINING | PT Referral |
| CONSULT / REFERRAL TO GAIT TRAINING | PT Referral |
| ACCUPRESSURE MASSAGE | CAM/integrative medicine |
| CONSULT / REFERRAL TO OSTEOPATHIC MANIPULATIVE THERAPY | CAM/integrative medicine |
| CONSULT / REFERRAL TO CHIROPRACTIC THERAPY | CAM/integrative medicine |
| CONSULT / REFERRAL FOR ACUPUNCTURE | CAM/integrative medicine |
| CONSULT / REFERRAL TO UPMC CENTER INTEGRATIVE MEDICINE | CAM/integrative medicine |
| CONSULT / REFERRAL TO MASSAGE THERAPY | CAM/integrative medicine |
| MASSAGE 60 MINUTES | CAM/integrative medicine |
| CONSULT / REFERRAL TO ORTHOPEDICS | Orthopedic referral |
| CONSULT / REFERRAL TO ORTHOPEDIC SURGERY | Orthopedic referral |
| CONSULT / REFERRAL TO PHYSICAL MEDICINE AND REHABILITATION | Pain management |
| CONSULT / REFERRAL TO PAIN CLINIC | Pain management |
| CONSULT / REFERRAL TO PAIN MANAGEMENT | Pain management |
| CONSULT / REFERRAL TO MEDICAL NUTRITION THERAPY | Lifestyle interventions |
| HOME EXERCISE PROGRAM | Lifestyle interventions |
| CONSULT / REFERRAL TO LIFESTYLE HEALTH COACHING PROGRAM | Lifestyle interventions |
| CONSULT / REFERRAL TO BEHAVIORAL HEALTH PROGRAM | Lifestyle interventions |
| BMI: DISCUSSED LIFESTYLE MODIFICATIONS | Lifestyle interventions |
| CONSULT / REFERRAL TO A PRACTICE-BASED WEIGHT MANAGEMENT PROGRAM | Lifestyle interventions |
| CONSULT / REFERRAL TO OSTEOARTHRITIS OF KNEE PROGRAM | Lifestyle interventions |
| WELLNESS GUIDE NC | Lifestyle interventions |
| CONSULT / REFERRAL TO MEDICAL NUTRITION THERAPY | Lifestyle interventions |

**Supplementary Table S3. KOA related medications identified from the Electronic Medical Records**

| **Generic Name of the Medication** | **Final Category Reported in Manuscript** |
| --- | --- |
| CAPSAICIN/MENTHOL | Topical analgesics |
| CAPSAICIN | Topical analgesics |
| CAPSAICIN/ME-SALICYLATE/MENTH | Topical analgesics |
| CAPSAICIN/CAMPHOR/MENTHOL | Topical analgesics |
| DICLOFENAC/MET SALICYL/MENTHOL | Topical analgesics |
| DICLOFENAC SODIUM | Topical analgesics |
| DICLOFENAC/CAPSICUM OLEORESIN | Topical analgesics |
| DICLOFENAC EPOLAMINE | Topical analgesics |
| DICLOFENAC SODIUM/CAPSAICIN | Topical analgesics |
| DICLOFENAC SODIUM/MENTHOL | Topical analgesics |
| KETOPROFEN | Topical analgesics |
| MELOXICAM/IRRIT.CNTR-IRR CMB 2 | Topical analgesics |
| IBUPROFEN | NSAIDs oral |
| NAPROXEN SODIUM | NSAIDs oral |
| DICLOFENAC SODIUM/MISOPROSTOL | NSAIDs oral |
| CELECOXIB | NSAIDs oral |
| SULINDAC | NSAIDs oral |
| DICLOFENAC SODIUM | NSAIDs oral |
| DICLOFENAC POTASSIUM | NSAIDs oral |
| DICLOFENAC SUBMICRONIZED | NSAIDs oral |
| ETODOLAC | NSAIDs oral |
| FLURBIPROFEN | NSAIDs oral |
| INDOMETHACIN | NSAIDs oral |
| KETOPROFEN | NSAIDs oral |
| KETOROLAC TROMETHAMINE | NSAIDs oral |
| MECLOFENAMATE SODIUM | NSAIDs oral |
| MELOXICAM | NSAIDs oral |
| MELOXICAM, SUBMICRONIZED | NSAIDs oral |
| NABUMETONE | NSAIDs oral |
| NAPROXEN | NSAIDs oral |
| NAPROXEN/ESOMEPRAZOLE MAG | NSAIDs oral |
| OXAPROZIN | NSAIDs oral |
| PIROXICAM | NSAIDs oral |
| TOLMETIN SODIUM | NSAIDs oral |
| TRAMADOL HCL/ACETAMINOPHEN | Tramadol |
| TRAMADOL HCL | Tramadol |
| MORPHINE SULFATE | Non-tramadol narcotic and combo analgesics |
| BUPRENORPHINE HCL | Non-tramadol narcotic and combo analgesics |
| OPIUM/BELLADONNA ALKALOIDS | Non-tramadol narcotic and combo analgesics |
| BUPRENORPHINE | Non-tramadol narcotic and combo analgesics |
| BUTORPHANOL TARTRATE | Non-tramadol narcotic and combo analgesics |
| CODEINE SULFATE | Non-tramadol narcotic and combo analgesics |
| HYDROMORPHONE HCL | Non-tramadol narcotic and combo analgesics |
| FENTANYL | Non-tramadol narcotic and combo analgesics |
| MORPHINE SULFATE/NALTREXONE | Non-tramadol narcotic and combo analgesics |
| FENTANYL CITRATE-0.9 % NACL/PF | Non-tramadol narcotic and combo analgesics |
| HYDROCODONE BITARTRATE | Non-tramadol narcotic and combo analgesics |
| HYDROMORPHONE HCL/PF | Non-tramadol narcotic and combo analgesics |
| HYDROMORPHONE HCL IN 0.9% NACL | Non-tramadol narcotic and combo analgesics |
| LEVORPHANOL TARTRATE | Non-tramadol narcotic and combo analgesics |
| MEPERIDINE HCL/PF | Non-tramadol narcotic and combo analgesics |
| MEPERIDINE HCL | Non-tramadol narcotic and combo analgesics |
| METHADONE HCL | Non-tramadol narcotic and combo analgesics |
| MORPHINE SULFATE/0.9% NACL/PF | Non-tramadol narcotic and combo analgesics |
| MORPHINE SULFATE/D5W | Non-tramadol narcotic and combo analgesics |
| MORPHINE SULFATE IN 0.9 % NACL | Non-tramadol narcotic and combo analgesics |
| TAPENTADOL HCL | Non-tramadol narcotic and combo analgesics |
| OXYMORPHONE HCL | Non-tramadol narcotic and combo analgesics |
| OXYCODONE HCL | Non-tramadol narcotic and combo analgesics |
| OXYCODONE MYRISTATE | Non-tramadol narcotic and combo analgesics |
| PROPOXYPHENE HCL | Non-tramadol narcotic and combo analgesics |
| TRAMADOL HCL | Non-tramadol narcotic and combo analgesics |
| ACETAMINOPHEN WITH CODEINE | Non-tramadol narcotic and combo analgesics |
| HYDROCODONE BITARTRATE/ASPIRIN | Non-tramadol narcotic and combo analgesics |
| OXYCODONE HCL/ACETAMINOPHEN | Non-tramadol narcotic and combo analgesics |
| HYDROCODONE/ACETAMINOPHEN | Non-tramadol narcotic and combo analgesics |
| HYDROCODONE/IBUPROFEN | Non-tramadol narcotic and combo analgesics |
| OXYCODONE HCL,TEREPHTH/ASPIRIN | Non-tramadol narcotic and combo analgesics |
| OXYCODONE HCL/ASPIRIN | Non-tramadol narcotic and combo analgesics |
| TRAMADOL HCL/ACETAMINOPHEN | Non-tramadol narcotic and combo analgesics |

**Supplementary Table S4. Knee OA procedures identified via HCPCS, ICD-10 PCS codes or CPT codes in the Electronic Medical Records**

| **Procedure Code** | **Description of the Code** | **Final Category Reported in Manuscript** |
| --- | --- | --- |
| J0702 | Injection, betamethasone acetate and betamethasone sodium phosphate, per 3 mg | Therapeutic injections |
| J0704 | Injection, betamethasone sodium phosphate, per 4 mg | Therapeutic injections |
| J0712 | Injection, cephapirin sodium, up to 500 mg | Therapeutic injections |
| J1020 | Injection, methylprednisolone acetate, 20 mg | Therapeutic injections |
| J1030 | Injection, methylprednisolone acetate, 40 mg | Therapeutic injections |
| J1040 | Injection, methylprednisolone acetate, 80 mg | Therapeutic injections |
| J1094 | Injection, dexamethasone acetate, 1 mg | Therapeutic injections |
| J1100 | Injection, dexamethasone sodium phosphate, 1 mg | Therapeutic injections |
| J1170 | Injection, hydromorphone, up to 4 mg | Therapeutic injections |
| J1200 | Injection, diphenhydramine HCl, up to 50 mg | Therapeutic injections |
| J1212 | Injection, dimethyl sulfoxide, 50%, 50 ml | Therapeutic injections |
| J1245 | Injection, dipyridamole, 10 mg | Therapeutic injections |
| J1300 | Injection, eculizumab, 10 mg | Therapeutic injections |
| J7318 | HYALURONAN OR DERIVATIVE, DUROLANE, FOR INTRA-ARTICULAR INJECTION, 1 MG | Therapeutic injections |
| J7320 | HYALURONAN OR DERIVITIVE, GENVISC 850, FOR INTRA-ARTICULAR INJECTION, 1 MG | Therapeutic injections |
| J7321 | HYALURONAN OR DERIVATIVE, HYALGAN, SUPARTZ OR VISCO-3, FOR INTRA-ARTICULAR INJECTION, PER DOSE | Therapeutic injections |
| J7322 | HYALURONAN OR DERIVATIVE, HYMOVIS, FOR INTRA-ARTICULAR INJECTION, 1 MG | Therapeutic injections |
| J7323 | HYALURONAN OR DERIVATIVE, EUFLEXXA, FOR INTRA-ARTICULAR INJECTION, PER DOSE | Therapeutic injections |
| J7324 | HYALURONAN OR DERIVATIVE, ORTHOVISC, FOR INTRA-ARTICULAR INJECTION, PER DOSE | Therapeutic injections |
| J7325 | HYALURONAN OR DERIVATIVE, SYNVISC OR SYNVISC-ONE, FOR INTRA-ARTICULAR INJECTION, 1 MG | Therapeutic injections |
| J7326 | HYALURONAN OR DERIVATIVE, GEL-ONE, FOR INTRA-ARTICULAR INJECTION, PER DOSE | Therapeutic injections |
| J7327 | HYALURONAN OR DERIVATIVE, MONOVISC, FOR INTRA-ARTICULAR INJECTION, PER DOSE | Therapeutic injections |
| J7328 | HYALURONAN OR DERIVATIVE, GELSYN-3, FOR INTRA-ARTICULAR INJECTION, 0.1 MG | Therapeutic injections |
| 20610 | ARTHROCENTESIS, ASPIRATION AND/OR INJECTION, MAJOR JOINT OR BURSA (EG, SHOULDER, HIP, KNEE, SUBACROMIAL BURSA) | Therapeutic injections |
| 27370 | INJECTION FOR KNEE ARTHROGRAM | Imaging Other |
| 27370 | INJECTION OF CONTRAST FOR KNEE ARTHROGRAPHY | Imaging Other |
| 73580 | ARTHROGRAM KNEE LEFT | Imaging Other |
| 73721 | MR JOINT LWR EXTR WITHOUT CONTRAST | Imaging Other |
| 73721 | MR JOINT LWR EXTR WITHOUT CONTRAST LEFT | Imaging Other |
| 73721 | MR JOINT LWR EXTR WITHOUT CONTRAST RIGHT | Imaging Other |
| 73722 | MR ARTHROGRAM LEFT LWR EXTR WITH CONTRAST VIA JOINT INJECTION | Imaging Other |
| 73722 | MR ARTHROGRAM RIGHT LWR EXTR WITH CONTRAST VIA JOINT INJECTION | Imaging Other |
| 73722 | MR JOINT LWR EXTR WITH CONTRAST LEFT | Imaging Other |
| 73722 | MR JOINT LWR EXTR WITH CONTRAST RIGHT | Imaging Other |
| 73723 | MR ARTHROGRAM LEFT LWR EXTR WITH AND WO CONTRAST VIA JOINT INJECTION | Imaging Other |
| 73723 | MR ARTHROGRAM RIGHT LWR EXTR WITH AND WO CONTRAST VIA JOINT INJECTION | Imaging Other |
| 73560 | XRAY KNEE 2 VIEWS LEFT | Imaging XRAY |
| 73560 | XRAY KNEE 2 VIEWS RIGHT | Imaging XRAY |
| 73562 | XRAY KNEE 3 VIEWS LEFT | Imaging XRAY |
| 73562 | XRAY KNEE 3 VIEWS RIGHT | Imaging XRAY |
| 73562 | XRAY KNEE 3+ VW | Imaging XRAY |
| 73564 | XRAY KNEE MINIMUM 4 VIEWS LEFT | Imaging XRAY |
| 73564 | XRAY KNEE MINIMUM 4 VIEWS RIGHT | Imaging XRAY |
| 73565 | XRAY KNEE BILAT STANDING | Imaging XRAY |
| 27310 | Arthrotomy, knee, with exploration, drainage, or removal of foreign body (eg, infection) | Knee surgery |
| 27331 | Arthrotomy, knee; including joint exploration, biopsy, or removal of loose or foreign bodies | Knee surgery |
| 27332 | Arthrotomy, with excision of semilunar cartilage (meniscectomy) knee; medial OR lateral | Knee surgery |
| 27333 | Arthrotomy, with excision of semilunar cartilage (meniscectomy) knee; medial AND lateral | Knee surgery |
| 27437 | ARTHROPLASTY PATELLA | Knee surgery |
| 27437 | Arthroplasty, patella; without prosthesis | Knee surgery |
| 27438 | Arthroplasty, patella; with prosthesis | Knee surgery |
| 27446 | Arthroplasty, knee, condyle and plateau; medial OR lateral compartment | Knee surgery |
| 27447 | Arthroplasty, knee, condyle and plateau; medial AND lateral compartments with or without patella resurfacing (total knee arthroplasty) | Knee surgery |
| 27550 | Closed treatment of knee dislocation; without anesthesia | Knee surgery |
| 27560 | Closed treatment of patellar dislocation; without anesthesia | Knee surgery |
| 29866 | Arthroscopy, knee, surgical; osteochondral autograft(s) (eg, mosaicplasty) (includes harvesting of the autograft[s]) | Knee surgery |
| 29867 | Arthroscopy, knee, surgical; osteochondral allograft (eg, mosaicplasty) | Knee surgery |
| 29870 | Arthroscopy, knee, diagnostic, with or without synovial biopsy (separate procedure) | Knee surgery |
| 29871 | Arthroscopy, knee, surgical; for infection, lavage and drainage | Knee surgery |
| 29873 | Arthroscopy, knee, surgical; with lateral release | Knee surgery |
| 29874 | Arthroscopy, knee, surgical; for removal of loose body or foreign body (eg, osteochondritis dissecans fragmentation, chondral fragmentation) | Knee surgery |
| 29875 | Arthroscopy, knee, surgical; synovectomy, limited (eg, plica or shelf resection) (separate procedure) | Knee surgery |
| 29876 | Arthroscopy, knee, surgical; synovectomy, major, 2 or more compartments (eg, medial or lateral) | Knee surgery |
| 29877 | Arthroscopy, knee, surgical; debridement/shaving of articular cartilage (chondroplasty) | Knee surgery |
| 29879 | Arthroscopy, knee, surgical; abrasion arthroplasty (includes chondroplasty where necessary) or multiple drilling or microfracture | Knee surgery |
| 29880 | Arthroscopy, knee, surgical; with meniscectomy (medial AND lateral, including any meniscal shaving) including debridement/shaving of articular cartilage (chondroplasty), same or separate compartment(s), when performed | Knee surgery |
| 29881 | Arthroscopy, knee, surgical; with meniscectomy (medial OR lateral, including any meniscal shaving) including debridement/shaving of articular cartilage (chondroplasty), same or separate compartment(s), when performed | Knee surgery |
| 29882 | Arthroscopy, knee, surgical; with meniscus repair (medial OR lateral) | Knee surgery |
| 29883 | Arthroscopy, knee, surgical; with meniscus repair (medial AND lateral) | Knee surgery |
| 29884 | Arthroscopy, knee, surgical; with lysis of adhesions, with or without manipulation (separate procedure) | Knee surgery |
| 0SHC04Z | Insertion of Internal Fixation Device into Right Knee Joint, Open Approach | Knee surgery |
| G0289 | Arthroscopy, knee, surgical, for removal of loose body, foreign body, debridement/shaving of articular cartilage (chondroplasty) at the time of other surgical knee arthroscopy in a different compartment of the same knee | Knee surgery |
| 27347 | Excision of lesion of meniscus or capsule (eg, cyst, ganglion), knee | Knee surgery |
| 27486 | Revision of total knee arthroplasty, with or without allograft; 1 component | Knee surgery |
| 0HRKX73 | Replacement of Right Lower Leg Skin with Autologous Tissue Substitute, Full Thickness, External Approach | Knee surgery |
| 0LBQ0ZZ | Excision of Right Knee Tendon, Open Approach | Knee surgery |
| 0LUQ0JZ | Supplement Right Knee Tendon with Synthetic Substitute, Open Approach | Knee surgery |
| 0LUR0JZ | Supplement Left Knee Tendon with Synthetic Substitute, Open Approach | Knee surgery |
| 0MBN0ZZ | Excision of Right Knee Bursa and Ligament, Open Approach | Knee surgery |
| 0QBD0ZZ | Excision of Right Patella, Open Approach | Knee surgery |
| 0QRB0JZ | Replacement of Right Lower Femur with Synthetic Substitute, Open Approach | Knee surgery |
| 0QRD0JZ | Replacement of Right Patella with Synthetic Substitute, Open Approach | Knee surgery |
| 0QUB07Z | Supplement Right Lower Femur with Autologous Tissue Substitute, Open Approach | Knee surgery |
| 0QUC07Z | Supplement Left Lower Femur with Autologous Tissue Substitute, Open Approach | Knee surgery |
| 0QUC0KZ | Supplement Left Lower Femur with Nonautologous Tissue Substitute, Open Approach | Knee surgery |
| 0QUF0JZ | Supplement Left Patella with Synthetic Substitute, Open Approach | Knee surgery |
| 0SBC0ZX | Excision of Right Knee Joint, Open Approach, Diagnostic | Knee surgery |
| 0SBC0ZZ | Excision of Right Knee Joint, Open Approach | Knee surgery |
| 0SBC4ZX | Excision of Right Knee Joint, Percutaneous Endoscopic Approach, Diagnostic | Knee surgery |
| 0SBC4ZZ | Excision of Right Knee Joint, Percutaneous Endoscopic Approach | Knee surgery |
| 0SBD4ZX | Excision of Left Knee Joint, Percutaneous Endoscopic Approach, Diagnostic | Knee surgery |
| 0SRC069 | Replacement of Right Knee Joint with Oxidized Zirconium on Polyethylene Synthetic Substitute, Cemented, Open Approach | Knee surgery |
| 0SRC0EZ | Replacement of Right Knee Joint with Articulating Spacer, Open Approach | Knee surgery |
| 0SRC0J9 | Replacement of Right Knee Joint with Synthetic Substitute, Cemented, Open Approach | Knee surgery |
| 0SRC0JA | Replacement of Right Knee Joint with Synthetic Substitute, Uncemented, Open Approach | Knee surgery |
| 0SRC0JZ | Replacement of Right Knee Joint with Synthetic Substitute, Open Approach | Knee surgery |
| 0SRC0L9 | Replacement of Right Knee Joint with Unicondylar Synthetic Substitute, Cemented, Open Approach | Knee surgery |
| 0SRC0LZ | Replacement of Right Knee Joint with Unicondylar Synthetic Substitute, Open Approach | Knee surgery |
| 0SRC0N9 | Replacement of Right Knee Joint with Patellofemoral Synthetic Substitute, Cemented, Open Approach | Knee surgery |
| 0SRC0NZ | Replacement of Right Knee Joint with Patellofemoral Synthetic Substitute, Open Approach | Knee surgery |
| 0SRD069 | Replacement of Left Knee Joint with Oxidized Zirconium on Polyethylene Synthetic Substitute, Cemented, Open Approach | Knee surgery |
| 0SRD06Z | Replacement of Left Knee Joint with Oxidized Zirconium on Polyethylene Synthetic Substitute, Open Approach | Knee surgery |
| 0SRD07Z | Replacement of Left Knee Joint with Autologous Tissue Substitute, Open Approach | Knee surgery |
| 0SRD0EZ | Replacement of Left Knee Joint with Articulating Spacer, Open Approach | Knee surgery |
| 0SRD0J9 | Replacement of Left Knee Joint with Synthetic Substitute, Cemented, Open Approach | Knee surgery |
| 0SRD0JA | Replacement of Left Knee Joint with Synthetic Substitute, Uncemented, Open Approach | Knee surgery |
| 0SRD0JZ | Replacement of Left Knee Joint with Synthetic Substitute, Open Approach | Knee surgery |
| 0SRD0L9 | Replacement of Left Knee Joint with Unicondylar Synthetic Substitute, Cemented, Open Approach | Knee surgery |
| 0SRD0LZ | Replacement of Left Knee Joint with Unicondylar Synthetic Substitute, Open Approach | Knee surgery |
| 0SRD0N9 | Replacement of Left Knee Joint with Patellofemoral Synthetic Substitute, Cemented, Open Approach | Knee surgery |
| 0SRD0NZ | Replacement of Left Knee Joint with Patellofemoral Synthetic Substitute, Open Approach | Knee surgery |
| 0SRT0J9 | Replacement of Right Knee Joint, Femoral Surface with Synthetic Substitute, Cemented, Open Approach | Knee surgery |
| 0SRT0JZ | Replacement of Right Knee Joint, Femoral Surface with Synthetic Substitute, Open Approach | Knee surgery |
| 0SRU0J9 | Replacement of Left Knee Joint, Femoral Surface with Synthetic Substitute, Cemented, Open Approach | Knee surgery |
| 0SRU0JA | Replacement of Left Knee Joint, Femoral Surface with Synthetic Substitute, Uncemented, Open Approach | Knee surgery |
| 0SRU0JZ | Replacement of Left Knee Joint, Femoral Surface with Synthetic Substitute, Open Approach | Knee surgery |
| 0SRV0J9 | Replacement of Right Knee Joint, Tibial Surface with Synthetic Substitute, Cemented, Open Approach | Knee surgery |
| 0SRV0JA | Replacement of Right Knee Joint, Tibial Surface with Synthetic Substitute, Uncemented, Open Approach | Knee surgery |
| 0SRV0JZ | Replacement of Right Knee Joint, Tibial Surface with Synthetic Substitute, Open Approach | Knee surgery |
| 0SRW0J9 | Replacement of Left Knee Joint, Tibial Surface with Synthetic Substitute, Cemented, Open Approach | Knee surgery |
| 0SRW0JA | Replacement of Left Knee Joint, Tibial Surface with Synthetic Substitute, Uncemented, Open Approach | Knee surgery |
| 0SRW0JZ | Replacement of Left Knee Joint, Tibial Surface with Synthetic Substitute, Open Approach | Knee surgery |
| 0SUC09C | Supplement Right Knee Joint with Liner, Patellar Surface, Open Approach | Knee surgery |
| 0SUC09Z | Supplement Right Knee Joint with Liner, Open Approach | Knee surgery |
| 0SUD09C | Supplement Left Knee Joint with Liner, Patellar Surface, Open Approach | Knee surgery |
| 0SUD09Z | Supplement Left Knee Joint with Liner, Open Approach | Knee surgery |
| 0SUT09Z | Supplement Right Knee Joint, Femoral Surface with Liner, Open Approach | Knee surgery |
| 0SUU09Z | Supplement Left Knee Joint, Femoral Surface with Liner, Open Approach | Knee surgery |
| 0SUV09Z | Supplement Right Knee Joint, Tibial Surface with Liner, Open Approach | Knee surgery |
| 0SUW09Z | Supplement Left Knee Joint, Tibial Surface with Liner, Open Approach | Knee surgery |
| 0SWC0JC | Revision of Synthetic Substitute in Right Knee Joint, Patellar Surface, Open Approach | Knee surgery |
| 0SWC0JZ | Revision of Synthetic Substitute in Right Knee Joint, Open Approach | Knee surgery |
| 0SWDXJC | Revision of Synthetic Substitute in Left Knee Joint, Patellar Surface, External Approach | Knee surgery |
| 0SWWXJZ | Revision of Synthetic Substitute in Left Knee Joint, Tibial Surface, External Approach | Knee surgery |
| 0YBF0ZZ | Excision of Right Knee Region, Open Approach | Knee surgery |
| 0YBF3ZX | Excision of Right Knee Region, Percutaneous Approach, Diagnostic | Knee surgery |
| 0YBG0ZZ | Excision of Left Knee Region, Open Approach | Knee surgery |
| 0YUB0KZ | Supplement Left Lower Extremity with Nonautologous Tissue Substitute, Open Approach | Knee surgery |

**Supplementary Table S5. Study variables mapped to the Consolidated framework of Implementation Research domains**

|  | **Consolidated framework of Implementation Research domains** | | | |
| --- | --- | --- | --- | --- |
|  | **Inner Setting** | **Outer Setting** | **Individual characteristics** | **Intervention** |
| **Explanatory Variables** |  |  |  |  |
| Patient-level | -- | Insurance coverage | Patient clinical information and demographics | -- |
| Physician -level | Volume of KOA cases seen | -- | Specialty | -- |
| Practice-level | Practice KOA volume | Location (Urban/Rural)  Number of licensed PTs at county level | -- | -- |
| **Outcome Variable** | -- | -- | -- | Presence or absence of an early PT referral |

**Supplementary Table S6. Number and percentage of patients that received an early PT referral by diagnostic category**

| **Patient diagnostic category** | **Number of patients identified** | **Number of patients that received PT referral** | **% that received PT referral** |
| --- | --- | --- | --- |
| KOA specific ICD10 codes (M17X) | 3712 | 604 | 16.74% |
| Knee symptom ICD10 codes (M24.56, M25.46, M25.66, M25.76) | 6123 | 1025 | 16.27% |

**Supplementary Table S7. Sensitivity Analysis for Early referral to PT: Subset of cohort with specific KOA diagnostic code (n=3712) at the index visit**

|  | Independent Variables |  | |
| --- | --- | --- | --- |
|  |  | **OR (95% CI)** | **p-value** |
|  | **Sex – Female (ref group: male)** | 1.26 (1.03, 1.53) | 0.024 |
|  | **Body Mass Index, kg/m^2^** |  |  |
|  | <25 (Ref Group) | 1.00 |  |
|  | ≥25 and <30 | 1.15 (0.83, 1.60) | 0.347 |
|  | ≥ 30 | 1.25 (0.92, 1.68) |  |
|  | Missing | 0.98 (0.63, 1.51) |  |
|  | **Pain category** |  |  |
|  | 0-3 (Ref group) | 1.00 | 0.023 |
|  | 4-6 | 0.93 (0.61, 1.43) |  |
|  | 7-10 | 0.82 (0.53, 1.25) |  |
|  | missing | 0.60 (0.40, 0.81) |  |
| **Practice Characteristics** | **PT availability >10/10,000 persons (ref group: <10 PTs per 10,000 persons)** | 2.03 (1.18, 3.47) | 0.011 |
|  | **Location, Rural (ref=urban)** | 0.55 (0.35, 0.87) | 0.011 |

**Supplementary Table S8. Sensitivity Analysis for Early referral to PT: Subset of cohort with only general knee symptoms codes (n = 6123) at the index visit**

|  | Independent Variables |  | |
| --- | --- | --- | --- |
|  |  | **OR (95% CI)** | **p-value** |
|  | **Sex – Female (ref group: male)** | 1.55 (1.33, 1.81) | <0.0001 |
|  | **Body Mass Index, kg/m^2^** |  |  |
|  | <25 (Ref Group) | 1.00 |  |
|  | ≥25 and <30 | 1.00 (0.79, 1.25) | 0.038 |
|  | ≥ 30 | 1.07 (0.86, 1.31) |  |
|  | Missing | 0.66 (0.47, 0.94) |  |
|  | **Pain category** |  |  |
|  | 0-3 (Ref group) | 1.00 | 0.001 |
|  | 4-6 | 0.65 (0.48, 0.89) |  |
|  | 7-10 | 0.65 (0.48, 0.88) |  |
|  | missing | 0.55 (0.41, 0.73) |  |
| **Practice Characteristics** | **PT availability >10/10,000 persons (ref group: <10 PTs per 10,000 persons)** | 1.33 (0.92, 1.94) | 0.132 |
|  | **Location, Rural (ref=urban)** | 0.74 (0.51, 1.06) | 0.101 |
